# A 9-mRNA signature measured from whole blood by a prototype PCR panel predicts 28-day mortality upon admission of critically ill COVID-19 patients

**DOI:** 10.3389/fimmu.2022.1022750

**Published:** 2022-11-01

**Authors:** Claire Tardiveau, Guillaume Monneret, Anne-Claire Lukaszewicz, Valérie Cheynet, Elisabeth Cerrato, Katia Imhoff, Estelle Peronnet, Maxime Bodinier, Louis Kreitmann, Sophie Blein, Jean-François Llitjos, Filippo Conti, Morgane Gossez, Marielle Buisson, Hodane Yonis, Martin Cour, Laurent Argaud, Marie-Charlotte Delignette, Florent Wallet, Frederic Dailler, Céline Monard, Karen Brengel-Pesce, Fabienne Venet, the RICO study group

**Affiliations:** ^1^ Joint Research Unit HCL-bioMérieux, Equipe d'Accueil (EA) 7426 “Pathophysiology of Injury-Induced Immunosuppression” (Université Claude Bernard Lyon 1 – Hospices Civils de Lyon, bioMérieux), Lyon, France; ^2^ Open Innovation and Partnerships (OIP), bioMérieux Société Anonyme (S.A.), Lyon, France; ^3^ Immunology Laboratory, Edouard Herriot Hospital – Hospices Civils de Lyon, Lyon, France; ^4^ Anaesthesia and Critical Care Medicine Department, Hospices Civils de Lyon, Edouard Herriot Hospital, Lyon, France; ^5^ Centre International de Recherche en Infectiologie (CIRI), INSERM U1111, CNRS, UMR5308, Ecole Normale supérieure de Lyon, Université Claude Bernard-Lyon 1, Lyon, France; ^6^ Centre d’Investigation Clinique de Lyon (CIC 1407 Inserm), Hospices Civils de Lyon, Lyon, France; ^7^ Medical Intensive Care Department, Hospices Civils de Lyon, Croix-Rousse Hospital, Lyon, France; ^8^ Medical Intensive Care Department, Hospices Civils de Lyon, Edouard Herriot Hospital, Lyon, France; ^9^ Anaesthesia and Critical Care Medicine Department, Hospices Civils de Lyon, Croix-Rousse Hospital, Lyon, France; ^10^ Medical Intensive Care Department, Hospices Civils de Lyon, Lyon sud Hospital, Pierre-Bénite, France; ^11^ Neurological Anesthesiology and Intensive Care Department, Hospices Civils de Lyon, Pierre Wertheimer Hospital, Lyon, France

**Keywords:** transcriptomic multiplex tool, SARS-CoV-2 infection, immune response, 28-day mortality prediction, personalized medicine

## Abstract

Immune responses affiliated with COVID-19 severity have been characterized and associated with deleterious outcomes. These approaches were mainly based on research tools not usable in routine clinical practice at the bedside. We observed that a multiplex transcriptomic panel prototype termed Immune Profiling Panel (IPP) could capture the dysregulation of immune responses of ICU COVID-19 patients at admission. Nine transcripts were associated with mortality in univariate analysis and this 9-mRNA signature remained significantly associated with mortality in a multivariate analysis that included age, SOFA and Charlson scores. Using a machine learning model with these 9 mRNA, we could predict the 28-day survival status with an Area Under the Receiver Operating Curve (AUROC) of 0.764. Interestingly, adding patients’ age to the model resulted in increased performance to predict the 28-day mortality (AUROC reaching 0.839). This prototype IPP demonstrated that such a tool, upon clinical/analytical validation and clearance by regulatory agencies could be used in clinical routine settings to quickly identify patients with higher risk of death requiring thus early aggressive intensive care.

## 1 Introduction

The current pandemic of coronavirus disease-2019 (COVID-19) caused by severe acute respiratory syndrome-related coronavirus 2 (SARS-CoV-2) has infected over 589 million patients with more than 6 million deaths worldwide as of August 2022. Disease severity is highly variable, with the vast majority of patients remaining asymptomatic or demonstrating minimal to mild symptoms such as fever, cough and shortness of breath. Nonetheless, it was reported that 5 to 10% of patients require intensive care due to rapid progression (9 to 12 days) toward acute respiratory distress syndrome (ARDS) requiring ICU (intensive care unit) admission and invasive mechanical ventilation (1, 2).

The immune response has been demonstrated to play a key role in the physiopathology of COVID-19. In the most severe phenotype, patients present a complex immune profile that evolves over time (3, 4). At ICU admission, their immune response is mostly characterized by altered immuno-inflammatory responses with inadequate response of type I interferons signaling and downregulation of IFN-stimulated genes (ISGs), increased cytokines levels (both pro- and anti-inflammatory), marked lymphopenia, elevated immature myeloid cells, and decreased monocyte HLA-DR (mHLA-DR). Those alterations were hypothesized to lead to microthrombosis and tissue injury, eventually resulting in ARDS, multiorgan failure and death (5). During the pandemic, many exploratory non-hypothesis-driven studies have been conducted for deciphering the immune processes. As a whole, these studies used mixed various flow approaches (spectral flow, multicolor flow, time of flight mass spectrometry), transcriptomic strategies (transcriptomic signatures, single-cell RNA-seq), functional testing, and multiplex measurement of soluble mediators. Results were mostly analyzed though multi-data/−omic approaches. While providing crucial information on COVID-19 pathophysiology, these approaches were mainly based on clinical research tools that are, due to several limitations (i.e., time consuming, lack of standardization, poorly reproducible between cohorts or costly), not usable in clinical routine at the patients’ bedside or central lab.

A prototype multiplex transcriptomic tool used on the BIOFIRE^®^ FILMARRAY^®^ System allows whole blood assessment of mRNA of several genes involved in various aspects of the immune response (6, 7). Thus, this Immune Profiling Panel (IPP) gene set could potentially contribute to better decipher the complex immune status of severe COVID-19 patients when admitted to ICU. Here, we investigated this transcriptomic prototype device in a large cohort of critically ill COVID-19 patients. We found that a set of 9-mRNA immune-related markers was capable in predicting 28-day mortality and providing relevant information about immune dysregulations.

## 2 Material and methods

### 2.1 Subject details

#### 2.1.1 RICO cohort

RICO (REA-IMMUNO-COVID) is an ongoing prospective observational clinical study. In this ancillary study, 309 patients were enrolled between August 2020 and August 2021 in five ICUs of university-affiliated hospitals (Hospices Civils de Lyon, Lyon, France). They all presented pulmonary infection with SARS-CoV-2. Results on this cohort have been published previously (8). Briefly, inclusion criteria were (1) man or woman ≥ 18 years of age (2), hospitalization in ICU for SARS-CoV-2 respiratory infection (3), first hospitalization in ICU (4), positive diagnosis of SARS-CoV-2 infection carried out by PCR or by another approved method in at least one respiratory sample (5), blood sampling in the first 24h after admission to ICU (Day 0) feasible and (6) patient or next of kin who has been informed of the terms of the study and has not objected to participating. Exclusion criteria were pregnancy, institutionalized patients and inability to obtain informed consent. In the present work, IPP was tested at Day 0. Patients were 65.0 years old [IQR, 57.0-72.0] and presented a disparate distribution of males and females (68/32).

The RICO study protocol was approved by ethics committee (Comité de Protection des Personnes Ile de France 1 – N°IRB/IORG #: IORG0009918) under agreement number 2020-A01079-30. This clinical study was registered at ClinicalTrials.gov (NCT04392401). The committee waived the need for written informed consent because the study was observational, with a low risk to patients, and no specific procedure, other than routine blood sampling, was required. The RICO cohort comply with the Declaration of Helsinki, principles of Good Clinical Practice and the French personal data protection act.

#### 2.1.2 Healthy donors

Concomitantly, blood samples from 49 healthy volunteers were independently obtained from EFS (Etablissement Français du Sang, Lyon, France). Briefly, healthy donors were 40 years old [IQR, 27-54] and were heterogeneously distributed between males and females (73/27). Samples were collected in April 2020 and November 2021.

We used the Etablissement Français du Sang standardized procedures for blood donation and followed provisions of articles R.1243–49 and the French public health code to obtain written non-opposition to the use of donated blood for research purposes from healthy volunteers. The blood donors’ personal data were deidentified before transfer to our research laboratory.

### 2.2 Method details

#### 2.2.1 Transcriptome analysis

Whole blood was collected in PAXgene™ tubes following the manufacturer’s guidelines. Briefly, samples were left for 2 hours at room temperature in contact with reagents in the tubes before being transferred to -20°C for at least 24 hours and stored at -80°C. Samples were run on the BIOFIRE^®^ FILMARRAY^®^ TORCH (BioFire Diagnostics^®^, USA) using the prototype IPP gene set. Results were delivered in less than an hour and normalized expression values of markers were computed and used for the analyses.

#### 2.2.2 Immunological markers measurements

CD3+ T cells count was performed on an automated volumetric flow cytometer (Aquios CL, Beckman Coulter). Standardized mHLA-DR values (AB/C, antibodies bound per cell) were obtained by flow cytometry (Navios, Beckman Coulter) with HLA-DR Quantibrite reagents (Becton Dickinson) as previously described (8).

### 2.3 Statistical analysis

The study cohort was split randomly in a 70/30 manner to obtain two datasets balanced on three parameters: age, sex and mortality. This resulted in a dataset of 216 patients used for machine learning training purposes and an independent test set of 93 patients used for performances validation. For datasets description, qualitative data were reported as counts and frequencies and quantitative data were reported as median [IQR range]. Clinical characteristics were compared with non-parametric Mann-Whitney-Wilcoxon test for continuous variables and a Fisher’s exact test or chi-squared test (as appropriate) for categorical variables. The level of significance was set at 5% two-sided tests. Statistical analyses were performed with R software version 3.6.2. Data were centered and scaled to perform non-supervised principal components analysis using FactoMineR package (version 2.4). Genes that were significantly associated with 28-day mortality in a univariate logistic regression model were used to build multivariate models for prediction of the 28-day survival status. Trained models were logistic regression with L1 (Lasso), L2 (Ridge) and mixed (ElasticNet) regularization, Partial Least Squares-discriminant (PLS) analysis and Support Vector Machines with linear kernels (linear SVM) using CARET package (version 6.0-84). To compensate the imbalanced repartition of mortality in our datasets, the Synthetic Minority Oversampling Technique (SMOTE) was applied for hyper parameters tuning (9). Models hyper parameters were chosen corresponding to the largest mean Area Under Precision Recall Curve (AUPRC) value among test folds from repeated cross-validation (k-fold=5, number of repeats=10) in the RICO training cohort (10) as sensitivity and Positive Predictive Values (PPV) are parameters of interest. Briefly, among the 5 machine learning algorithm evaluated, the hyper parameters selected were as follow, Lasso (α=1, λ=0.031), Ridge (α=0, λ=0.556), ElasticNet (α=0.35, λ=0.37), PLS (ncomp=1) and linear SVM (C=0.367). AUPRC and their bootstrap 95% confidence interval was obtained using PRROC (version 1.3.1) and boot (version 1.3-28) packages. Number of bootstrap resamples has been set to N=1000. Variables relative importance in the linear SVM model was calculated using the FIRM method from vip package (version 0.3.2) (11). Area Under the ROC Curve (AUROC) and bootstrap 95% confidence interval and diagnostic performances (sensitivity, specificity, positive and negative predictive values and F1 score) at optimal cut-offs for the 9-mRNA panel as well as others individual parameters were obtained considering respective Youden values from cutpointr package (version 1.1.1) defined on training dataset and then applied on test dataset values. The F1 score (harmonic mean of recall and precision) was used as model accuracy measure due to unbalanced data. The survival probability rendered by the linear SVM machine learning model with the 9 genes significantly associated with 28-day mortality was tested in a multivariate logistic regression analysis with confounding clinical factors found to be significantly associated to 28-day mortality in the univariate analysis (i.e. age, Charlson and SOFA scores). The SAPS II score was excluded from the analysis as age and severity were captured by age and SOFA score. As the number of events of interest in the combined train and test cohorts was 52; the inclusion of three confounding factors in such multivariate analysis appeared appropriate.

## 3 Results

### 3.1 Clinical characteristics at ICU admission

Patient characteristics (whole, training and test cohorts) are shown in **Table 1**. A total of 309 patients were hospitalized in 5 hospitals in Lyon between August 2020 and August 2021. Briefly and as previously reported, we observed that 70% of patients were male. Overall, patient characteristics were similar to those described in previously published cohorts of critically ill COVID-19 patients. Patients were admitted to the Intensive Care Unit (ICU) with a median of 9 days after presentation of the first symptoms [IQR, 6.0-11.0]. They presented comorbidities as assessed by their Charlson score. Among these comorbidities, diabetes was preponderant (31%). As reported worldwide, patients presented a high median BMI (kg/m3) of 29.1 [IQR, 26.1-33.2]. In terms of severity of the disease, patients presented a decreased PaO2/FiO2 (mmHg) with a median of 97.5 [IQR: 74.3-146.5], elevated SOFA [median: 2.0; IQR: 1.0-5.0] and SAPS II scores [median: 30.0; IQR: 23.5-39.0]. At admission, 17.2% patients required invasive mechanical ventilation. All patients were under systemic corticoid therapy upon or at admission (6mg dexamethasone daily). Patients spent a median 18 days [IQR, 11.0-31.8] in the hospital among which 8 days [IQR, 4.0-17.0] were spent in the ICU. About a third developed secondary infections. Most of these were pneumopathies (87/99) among which 16 were fungal infections. Lastly, among the 309 patients, 52 (17%) died by day 28.

**Table 1:** Clinical characteristics of critically ill patients with COVID-19.

	All patients(n = 309)	Training			Test		
		28-day survivors (n = 179)	28-day non survivors (n = 37)	*p* value	28-day survivors (n = 78)	28-day non survivors (n = 15)	*p* value
**Demographics**							
Age - years	65.0 [57.0-72.0]	64.0 [55.0-70.0]	71.0 [69.0-78.0]	**<0.001**	64.5 [55.0-70.0]	72.0 [68.0-76.0]	**<0.001**
Male gender – *n (%)*	210 (68.0%)	119 (66.5%)	28 (75.7%)	0.369	52 (66.7%)	11 (73.3%)	0.767
Body mass index - kg/m²	29.1 [26.1-33.2]	29.1 [25.7-33.1]	29.6 [27.33-33.0]	0.674	28.7 [26.1-33.4]	30.1 [28.0-33.2]	0.335
BMI > 30 – *n (%)*	128 (43.7%)	75 (43.9%)	15 (46.9%)	0.903	30 (40.0%)	8 (53.3%)	0.504
**Comorbidities**							
Diabetes: none - *n (%)*	213 (69%)	131 (73.2%)	18 (48.7%)	**0.001**	55 (70.5%)	9 (60.0%)	0.534
Diabetes: with damage - *n (%)*	15 (4.9%)	6 (3.4%)	6 (16.2%)		3 (3.9%)	0 (0.0%)	
Diabetes: w/o organic damage - *n (%)*	81 (26.2%)	42 (23.4%)	13 (35.1%)		20 (25.6%)	6 (40.0%)	
Charlson Score - points	1.0 [0.0-2.0]	1.0 [0.0-1.0]	2.0 [1.0-4.0]	**<0.001**	0.5 [0.0-1.0]	1.0 [1.0-2.0]	**0.043**
**Clinical severity at admission**							
Delay between symptoms and ICU admission - days	9.0 [6.0-11.0]	9.0 [7.0-12.0]	7.5 [5.0-9.8]	**0.013**	9.0 [6.8-10.3]	6.0 [5.5-9.0]	**0.041**
ARDS at admission – *n (%)*	72 (23.8%)	41 (23.3%)	11 (30.6%)	0.478	13 (17.1%)	7 (46.7%)	**0.029**
SOFA Score - points	2.0 [1.0-5.0]	2.0 [0.0-5.0]	4.0 [2.0-6.0]	**0.008**	2.0 [0.3-3.0]	3.0 [1.5-7.5]	0.068
SAPS II Score - points	30.0 [23.5-39.0]	30.0 [23.0-38.8]	39.0 [33.0-47.0]	**<0.001**	27.5 [21.0-34.0]	32.0 [26.8-41.3]	0.086
PaO2/FIO2 - mmHg	97.5 [74.3-146.5]	95.0 [77.5-146.0]	82.0 [70.5-147.8]	0.376	98.0 [89.0-149.0]	104.5 [93.8-128.3]	0.844
pH	7.45 [7.42-7.49]	7.46 [7.42-7.49]	7.44 [7.40-7.49]	0.591	7.46 [7.43-7.49]	7.47 [7.40-7.49]	0.769
Lactate - mmol/L	1.65 [1.30-2.00]	1.70 [1.37-2.02]	1.90 [1.40-2.20]	0.326	1.50 [1.30-1.90]	1.40 [1.30-1.80]	0.785
**Organ support**							
Invasive mechanical ventilation at Day 0 – *n (%)*	53 (17.2%)	29 (16.2%)	10 (27%)	0.186	9 (11.5%)	5 (33.3%)	**0.046**
Vasoactive drugs - *n (%)*	35 (11.4%)	19 (10.7%)	8 (21.6%)	0.120	6 (7.7%)	2 (13.3%)	0.611
Renal replacement therapy - *n (%)*	31 (10.0%)	13 (7.3%)	11 (29.7%)	**<0.001**	4 (5.1%)	3 (20.0%)	0.080
**Follow-up**							
MV duration - days	14.0 [7.0-27.3]	17.0 [7.0-34.0]	12.0 [7.0-20.0]	0.110	22.5 [11.3-30.8]	12.0 [6.5-15.5]	**0.030**
ICU length of stay - days	8.0 [4.0-17.0]	8.0 [3.0-16.0]	12.0 [8.0-19.0]	**0.017**	8.0 [5.0-16.8]	11.0 [5.5-15.5]	0.871
Hospital length of stay - days	18.0 [11.0-31.8]	18.0 [10.0-36.5]	15.0 [9.0-21.0]	**0.024**	20.5 [13.0-34.8]	14.0 [7.5-18.5]	**0.014**
28-day mortality - *n (%)*	52 (16.8%)	0 (0%)	37 (100%)	**<0.001**	0 (0%)	15 (100%)	**<0.001**
90-day mortality - *n (%)*	66 (21.9%)	12 (6.8%)	37 (100%)	**<0.001**	2 (2.7%)	15 (100%)	**<0.001**
ICU-acquired infections – *n (%)*	99 (33.1%)	55 (31.6%)	19 (54.3%)	**0.018**	15 (20.0%)	10 (66.7%)	**<0.001**
ICU-acquired pneumopathies - *n (% IAI)*	87/99 (87.9%)	48/55 (87.3%)	18/19 (94.7%)	0.366	12/15 (80.0%)	9/10 (90.0%)	0.504
**Immunological parameters at admission**							
mHLA-DR – AB/C	8950.0 [6655.5-12173.5]	9246.0 [6770.0-12827.0]	7377.5 [4760.8-11413.0]	**0.029**	8939.0 [6859.8-11038.8	8967.0 [7551.5-11118.0]	0.810
CD3 T cells – absolute count	325.0 [228.0-505.5]	326.0 [236.5-506.0]	303.0 [200.0-400.0]	0.056	326.0 [218.0-515.0]	500.0 [251.0-555.5]	0.583

Medians and interquartile ranges [Q1-Q3] are shown for continuous variables or numbers and percentages are presented for categorical variables. COVID-19 patients were separated in two groups based on their 28-day survival status after admission. Sequential organ failure (SOFA) and simplified acute physiology II (SAPS II) scores were calculated during the first 24 hours after admission. Acute respiratory distress at admission was based on the Berlin definition (12). Data were compared using the nonparametric Mann-Whitney-Wilcoxon test for continuous variables or the chi-square/Fisher exact test for categorical variables.

p values ≤ 0.05 are highlighted in bold.

### 3.2 Cellular immunology and IPP transcriptomic profile

As previously reported, mHLA-DR was decreased with a median of 8950 AB/C [IQR, 6655.5-12173.5] in comparison with references values (> 13 500 AB/C) and patients presented with severe lymphopenia with a median T cell count of 325 cells/µL [IQR, 228.0-505.5] compared to reference values > 1000 cells/µL (13, 14).

**Figure 1:**
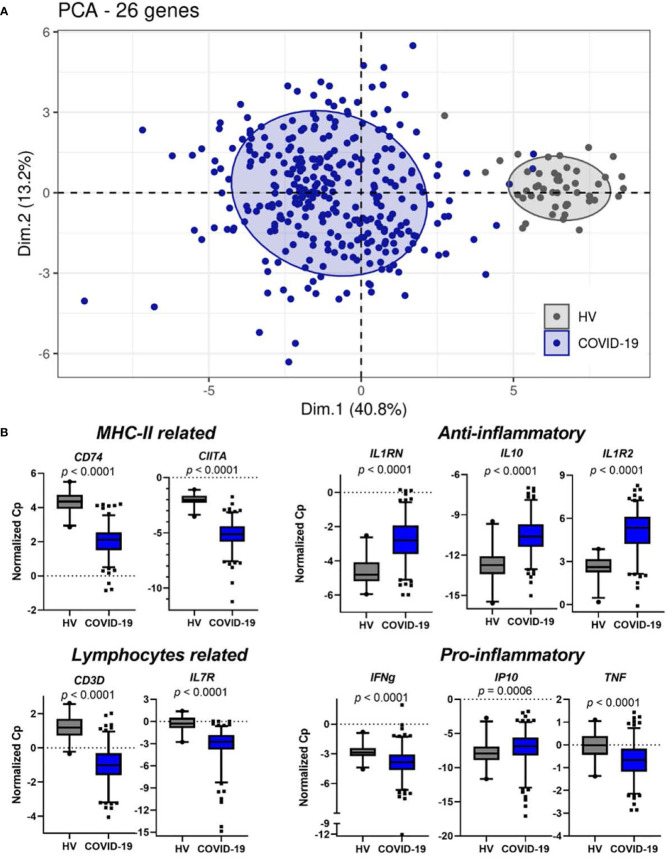
IPP markers distinguish healthy donors from critically ill COVID-19 patients and are associated with immunological parameters. **(A)** Non-supervised PCA on IPP markers measured at admission (Day 0) in critically ill COVID-19 patients (n=309) and healthy donors (n=49). **(B)** Boxplots representation of the expression of IPP markers related to immunological parameters. p values were computed with a Mann-Whitney-Wilcoxon test. Whiskers indicate the 2.5 and 97.5 percentiles.

Non-supervised clustering using principal component analysis (PCA) on the 26 IPP mRNA transcripts resulted in clear distinct clusters between the 309 critically ill COVID-19 patients at admission and 49 healthy donors (**Figure 1A**). The IPP gene set revealed significant changes in inflammation and cellular-associated transcriptomic markers in COVID-19 patients when compared with healthy volunteers. *CD74, CIITA, CD3D* and *IL7R* were downregulated in critically ill COVID-19 patients (**Figure 1B**) in accordance with the occurrence of altered monocyte and T lymphocyte responses. Pro- and anti-inflammatory responses (e.g. *IL1RN, IL10, IL1R2* and *IP10*) were both upregulated in patients (**Figure 1B**). In contrast, we observed that *IFNG* and *TNF* mRNA levels were lower in patients than in healthy donors. Overall, these first results indicated that the IPP gene set provided relevant information recapitulating immune dysregulation known in COVID-19 critically ill patients, in line with the vast literature previously published on this population.

### 3.3 Association with 28-day mortality

**Figure 2:**
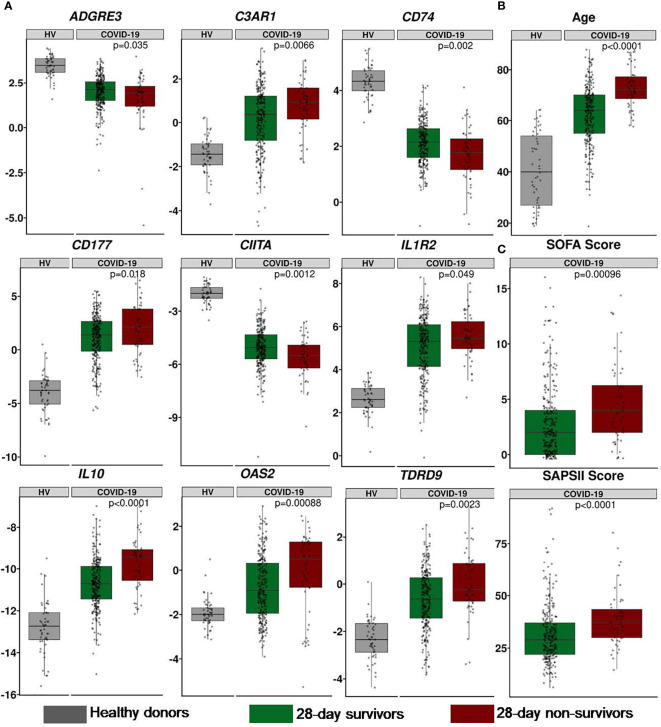
Description of IPP 9-mRNA and clinical parameters in the RICO cohort of 309 critically ill COVID-19 patients. **(A)** Expression of IPP markers (values are presented as normalized Cp). **(B)** Age (years). **(C)** Clinical scores. All parameters are presented at admission between 28-day survivors (green) and non-survivors (red). When relevant, reference values of healthy donors are presented in grey. The p-value were generated using a Mann-Whitney-Wilcoxon test between survivors and non-survivors.

We further investigated the association between IPP markers and the 28-day mortality. A logistic regression univariate analysis was performed on the training dataset composed of 216 patients, we identified 9 genes that were significantly associated with 28-day mortality (**Table 2**), namely *ADGRE3, C3AR1, CD177, CD74, CIITA, IL10, IL1R2, OAS2* and *TDRD9.* Among those, *ADGRE3, CD74* and *CIITA* were significantly downregulated in non-survivors when compared to survivors, while all other markers were upregulated in non-survivors. Among cellular parameters, only CD3 T cells count was significantly associated with 28-day mortality in a logistic regression model (*p*=0.031). Descriptive boxplots of the 9 genes that compose the panel along with clinical scores and age regarding with 28-day survival status are presented in **Figure 2**. Consistent with the existing literature, age, SOFA, SAPS II and Charlson scores were significantly associated with mortality in univariate analysis.

**Table 2:** Association between IPP transcriptomic, immune, clinical or demographical parameters and 28-day survival status: univariate analyses.

	OR_IQR_ [95% CI]	IQR	*p* value
ADGRE3	0.66 [0.45-0.93]	1.17	**0.021**
C3AR1	2.02 [1.20-3.55]	1.90	**0.010**
CD177	1.76 [1.10-2.94]	2.69	**0.022**
CD74	0.56 [0.34-0.88]	1.08	**0.014**
CIITA	0.54 [0.33-0.82]	1.41	**0.005**
IL10	2.88 [1.72-5.01]	1.67	**< 0.001**
IL1R2	2.82 [1.15-3.39]	1.84	**0.016**
OAS2	1.82 [1.02-3.31]	2.43	**0.045**
TDRD9	1.86 [1.15-3.11]	1.60	**0.014**
mHLA-DR [antibody/cell]	0.97 [0.67-1.30]	6246	0.856
CD3 T Cells [cells/µL]	0.56 [0.31-0.90]	258.5	**0.031**
SOFA Score	1.50 [1.04-2.15]	4	**0.029**
SAPS II Score	1.65 [1.17-2.33]	16	**0.004**
Charlson Score	1.62 [1.14-2.42]	2	**0.017**
Age	4.95 [2.62-10.17]	15.25	**< 0.001**

Two hundred and sixteen critically ill patients were included in the training set. One hundred and seventy-nine patients survived up until Day 28 and thirty-seven died. The association between 28-day survival status and transcriptomic IPP parameters, classical immune, clinical or demographical parameters were performed by implementing univariate logistic regression models. To allow comparison between models, odds ratios calculated for each parameter were normalized to an increment from first to third quartile (inter quartile range odd ratios, OR_IQR_). p values ≤ 0.05 are highlighted in bold.

**Figure 3:**
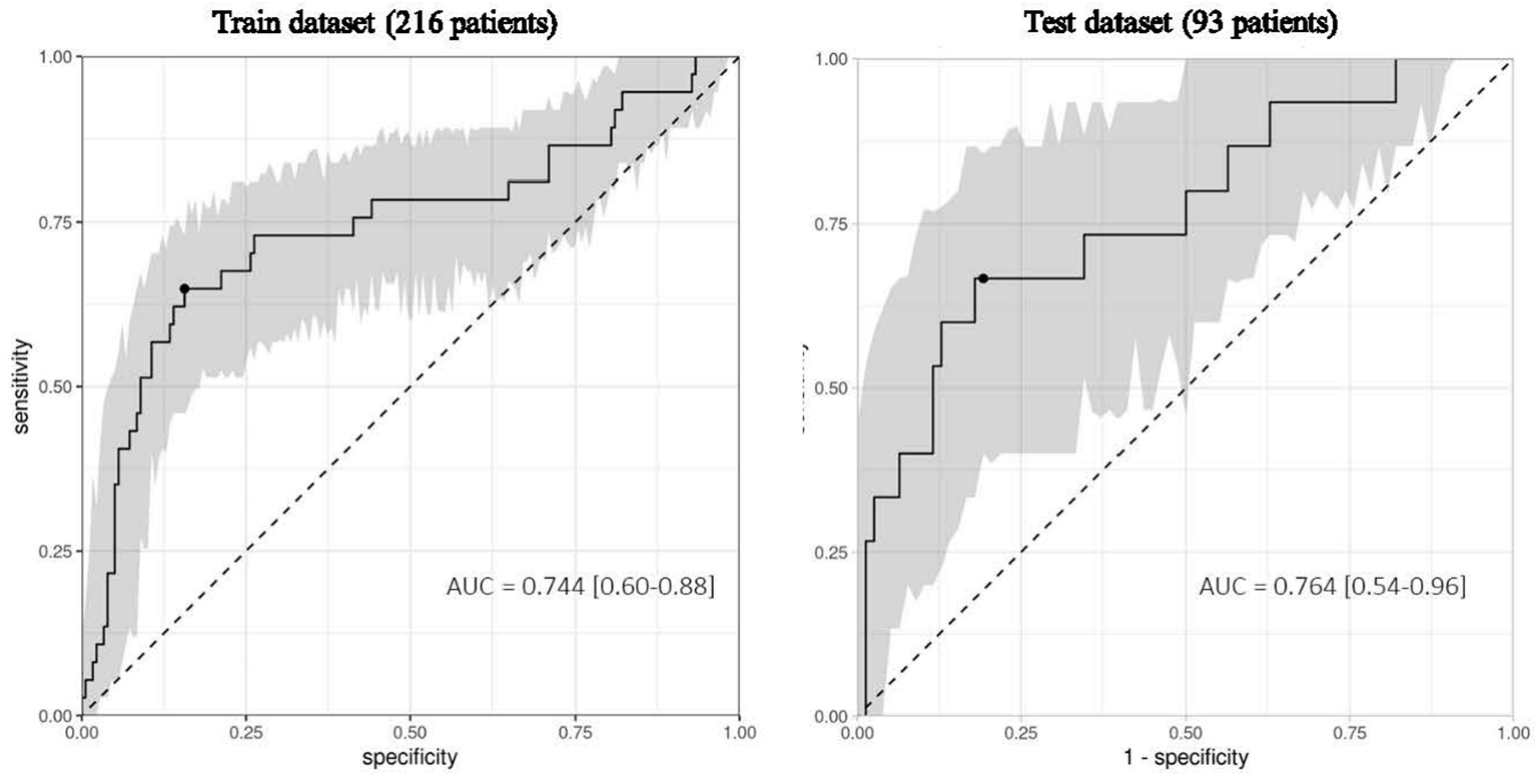
IPP markers measured at admission predict 28-day mortality in critically ill COVID-19 patients. Area Under the Receiver Operating Characteristics curve (AUC) calculated on the training dataset of 216 patients and the independent test set of 93 critically ill COVID-19 patients using the 9-mRNA panel at their admission in the ICU. The 95% confidence interval (grey) was calculated using bootstrap with 1000 repetitions.

This 9-mRNA signature was then used in five different machine learning models to predict the 28-day survival status in the training dataset. We selected the best performing machine learning model based on the area under the precision-recall curve (**Table 3**). We found that the linear support vector machine learning model presented the best AUPRC (0.431) and second best AUROC (0.744). We then tested the tuned models on the test dataset (93 patients) to confirm results obtained in training dataset. The AUPRC calculated was 0.431 while the AUROC reached 0.764 (**Table 3**). The ROC curves generated on the training and test datasets are shown in **Figure 3**. Overall, results from the test dataset confirm the robustness of those obtained on the training dataset to predict mortality. The 9-mRNA signature was further tested in a logistic regression multivariate analysis with the following confounding factors: age, SOFA score and Charlson score. The signature remained significantly associated with 28-day mortality with an odds ratio per interquartile range of 3.78 (**Table 4**).

**Table 3:** Summary of the performance of the five different machine learning models on the training and test datasets.

ML Models	AUROC_training_[95% CI]	AUPRC_training_[95% CI]	AUROC_test_[95% CI]	AUPRC_test_[95% CI]
Elastic Net	0.715 [0.575-0.844]	0.361 [0.243-0.524]	0.721 [0.493-0.938]	0.380 [0.171-0.662]
Ridge	0.737 [0.612-0.859]	0.406 [0.257-0.584]	0.751 [0.575-0.927]	0.326 [0.164-0.558]
Lasso	0.754 [0.630-0.874]	0.402 [0.256-0.576]	0.748 [0.554-0.932]	0.346 [0.168-0.620]
PLS	0.732 [0.605-0.853]	0.406 [0.256-0.579]	0.744 [0.567-0.924]	0.312 [0.156-0.566]
**svmLin**	**0.744** **[0.600-0.881]**	**0.431** **[0.278-0.610]**	**0.764** **[0.536-0.960]**	**0.431** **[0.214-0.720]**

Area Under the Receiver Operating Characteristics Curves and Area Under the Precision Recall Curves with their respective 95% confidence intervals calculated for 5 different machine learning models on the training and test datasets to predict 28-day survival. Performances of the best model are highlighted in bold.

**Table 4:** Association between 9-mRNA signature, clinical or demographical parameters and 28-day survival status: multivariate analysis.

	OR_IQR_ [95% CI]	IQR	*p* value
9-mRNA signature	3.78 [2.22-6.73]	0.26	**< 0.001**
SOFA Score	1.34 [0.90-2.03]	4	0.151
Charlson Score	1.37 [1.04-1.81]	2	**0.015**
Age	5.11 [2.82-9.96]	15	**< 0.001**

Three hundred and nine critically ill patients were included. Fifty two patients died by Day 28. The association between 28-day survival status and the 9-mRNA signature and clinical and demographical parameters was evaluated by implementing multivariate logistic regression models with the following confounding factors: age, SOFA score and Charlson score. To allow comparison between models, odds ratios calculated for each parameter were normalized to an increment from first to third quartile (inter quartile range odd ratios, OR_IQR_). p values ≤ 0.05 are highlighted in bold.

**Figure 4:**
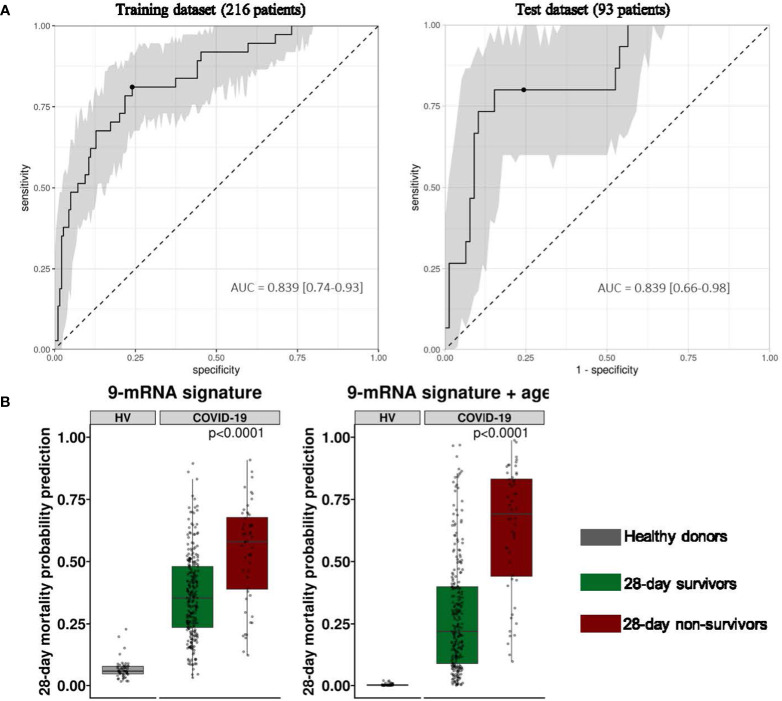
Performance of the 9-mRNA signature combined with age to predict 28-day survival status in critically ill COVID-19 patients at ICU admission. **(A)** Area Under the Receiver Operating Characteristics curve (AUC) calculated on the training dataset of 216 patients and the independent test set of 93 critically ill COVID-19 patients using the 9-mRNA panel along with the age at their admission in ICU. The 95% confidence interval (grey) is calculated using bootstrap with 1000 repetitions. **(B)** Probability of 28-day mortality from linear SVM model trained on 9-gene panel (left) and 9-gene panel combined with age (right) on the entire cohort (n=309) of patients and healthy donors (n=49). p-values were generated using a Mann-Whitney-Wilcoxon test between survivors and non-survivors.

In order to test the added value of a 9-mRNA signature, we then examined individual 28-day mortality prediction performance of each mRNA in the signature set as well as age, SOFA and SAPS II scores, and T cell count using logistic regression models. We found that the 9-mRNA signature presented the best AUPRC (and AUROC) when compared to all other individual parameters. Using the Youden threshold, we calculated the sensitivity, specificity, positive predictive value, negative predictive value and F1 score for each parameter (**Table 5**). Not surprisingly, we found that age was also well associated with 28-day mortality, which is consistent with numerous observations made since the beginning of the pandemic. Based on this, we next investigated whether a machine learning model including age and the 9-mRNA signature could be more informative to predict 28-day mortality. Using the same previous methodology, we found that the linear support vector machine learning model composed of the 9 genes and age was the best to predict 28-day mortality with an AUPRC of 0.539 (AUROC = 0.839) in the training dataset. The test dataset provided similar results, i.e., an AUPRC of 0.532 (AUROC = 0.839) (**Figure 4A**). Results from the two models (i.e., with and without age) are depicted in **Figure 4B**.

**Table 5:** Indicators of 28-day survival prediction performance of individual transcripts and the 9-mRNA panel along with age, CD3 T cells count, SOFA and SAPS II scores at admission.

Parameters	AUROC_Test_	AUPRC_Test_	Sensitivity	Specificity	PPV	NPV	F1 Score
**9-mRNA signature**	**0.764**	**0.431**	**0.667**	**0.808**	**0.400**	**0.926**	**0.500**
Age	0.763	0.429	0.733	0.667	0.297	0.929	0.423
*CD177*	0.606	0.352	0.467	0.705	0.233	0.873	0.311
*IL10*	0.717	0.351	0.533	0.872	0.444	0.907	0.485
SOFA Score	0.647	0.339	0.533	0.654	0.229	0.879	0.320
*OAS2*	0.305	0.270	0.067	0.949	0.200	0.841	0.100
*TDRD9*	0.668	0.266	0.667	0.513	0.208	0.889	0.317
SAPSII Score	0.645	0.222	0.500	0.718	0.241	0.889	0.326
#CD3 T cells	0.455	0.180	0.467	0.338	0.121	0.765	0.192
*C3AR1*	0.578	0.172	0.600	0.590	0.220	0.885	0.321
*IL1R2*	0.541	0.160	0.800	0.346	0.190	0.900	0.308
*CD74*	0.697	0.133	0.400	0.833	0.316	0.878	0.353
*ADGRE3*	0.594	0.126	0.667	0.487	0.200	0.884	0.308
*CIITA*	0.644	0.118	0.400	0.782	0.261	0.871	0.316

Parameters are presented by descending Area Under the Precision Recall Curve (AUPRC) values. Values superior to 0.75 for Area Under the Receiver Operating Characteristics curve (AUC) and 0.4 for AUPRC are underlined.

Prediction performances of 9-mRNA signature are highlighted in bold.

## 4 Discussion

Clinical presentation of COVID-19 ranges from asymptomatic, mild infection to severe cases with acute respiratory distress syndrome, respiratory failure and, ultimately, death. It is now well established that immune alterations play a pivotal role in determining the severity of the disease course (15). Finding effective patient-tailored care management for COVID-19 patients that take into account their immune status is key to lessening the clinical burden and improve prognosis (16). Different approaches have been used to characterize the immune status in COVID-19 patients at the protein (circulating cytokines and/or other biomarkers), cellular (characterization of the immune subsets and functionality) or RNA levels (bulk/single-cell RNA-seq in whole blood, respiratory fluids) (3, 5, 17–19). Many groups over the past 2 years have worked on the identification of risk factors for severe disease progression in order to identify patients at high-risk of evolving towards a severe outcome. Among illustrative examples, in a study combining ~50 clinical features and ~200 high-dimensionality immunological features, Mathew et al. previously reported three distinct immunotypes associated with COVID-19 severity (20). In an extensive immune assessment study combining cellular data accessed by flow cytometry, soluble immune markers (multiplex cytokine analysis), RNA expressions (Nanostring) and serology (ELISA), Laing et al. identified a core peripheral blood immune signature in COVID-19 patients, which could identify settings of immunopathology, correlate with disease severity and anticipate clinical progression (4). In another elegant work, Abers et al. established that longitudinal trajectories of 11 immune-based circulating biomarkers were substantially associated with mortality when increased (10) or decreased (1) providing additional evidence that immune-based biomarkers may provide an early warning of COVID-19 outcome (21). Transcriptomic approaches have shown that they could discriminate between distinct physio-pathological states of the COVID-19 (e.g paucisymptomatic, mild/moderate and severe) (22). Recently, a 6-gene signature was identified to predict COVID-19 mortality based on cohort explorative approaches (23).

However, to date, none is implemented in the standard bundle of care of patients. The IPP prototype tool measures immune-related markers that were pragmatically selected based on their known-documented function or prognostic significance with the aim to assess the immune status of sepsis patients in a multi-dimensional way (7), recently it was demonstrated to predict 30-day mortality in patients with sepsis (24). In addition, technically speaking, the IPP prototype device could be used with its dedicated measurement platform to provide results in less than an hour from whole blood. When compared to other devices used for transcriptomic analyses, the IPP tool presents with several advantages, as it does not require any specific technicity. It is easy to use as it works with whole blood directly instead of extracted RNAs. Thus, the IPP prototype device presents the potential, in the future, to be used as a very appropriate and practical tool for implementation at the bedside.

In this study, we showed that IPP captured immune response dysregulations induced by SARS-CoV-2 infection. For examples, monocyte alterations (CD74, CIITA mRNA), lymphopenia (CD3, IL7R mRNA), increased anti-inflammatory response (IL10, IL1RN mRNA), altered IFN response (OAS2, IFNG mRNA) were observed.

Most importantly, from this whole blood multiplex mRNA assessment, we reported that the IPP prototype resulted in prediction of 28-day survival with a sensitivity of 0.667 and specificity of 0.808. We found that 9 genes associated with 28-day mortality and using machine learning approaches. This 9-mRNA signature could be used to predict 28-day survival status. Among them, some were already known and described in the literature for their role in pathophysiology and/or association with mortality such as IL10, CD74, CTIIA, IL1R2, CD177 and C3AR1 (22, 25–28). For example, increased expressions of IL10, IL1R2 mRNA and decreased expression of CD74 and CIITA mRNA were described in monocytes of progressive COVID-19 patients compared with stable patients suggesting the acquisition of a regulatory phenotype by myeloid cells. In the same study, increased expression of IL1R2 was also observed in neutrophils (29). Regarding neutrophils, the increased expressions of CD177, IL1R2 and S100A9 in our study agree with the existing literature, which points towards the induction of a dysregulated neutrophil function in COVID-19 patients with increased NET production that aggravates the pathophysiology of COVID-19. Similarly, increased mRNA expression of IL10 was reported in regulatory T cells of severe COVID-19 patients suggesting a defective adaptive immune response (30). Saichi et al. reported that antigen-presenting cells from severe COVID-19 patients presented with defects in several antiviral processes among which a downregulation of MHC class II related genes was observed in both monocytes and dendritic cells (31). Thus, while we cannot discriminate the cell specific mRNA deregulation in our study, we believe that the current results are consistent with the literature and thus that the fully automated IPP transcriptomic solution can capture immune deregulation induced by COVID-19 in the most severe patients.

In addition, we observed that OAS2 was also associated with mortality and its expression level at admission was informative to build our 9-mRNA model. OAS2 is an interferon stimulated gene involved in interferon response, it was previously reported to be associated with COVID-19 severity (32). Nonetheless, the current literature is conflicting regarding the OAS2 role in the pathophysiology of COVID-19. Indeed, on the one hand, a haplotype in the region containing OAS2 has been described to be protective against severe COVID-19 (33). on the other hand, transcriptomic levels of OAS2 in PBMCs were found to be upregulated in severe cases of COVID-19 (34, 35). TDRD9 and ADGRE3 were chosen since they have been previously demonstrated to be part of the SRS1 signature in sepsis (36). In agreement, although their precise role in pathophysiology remains to be further explored, both mRNAs were found to be associated with 28-day mortality in COVID-19 critically ill patients.

Most importantly, beyond the individual predictive value of the 9 mRNAs, their combination, based on machine learning models, was found to be a robust indicator of 28-day mortality within an AUROC of 0.764. Previous studies have demonstrated that various combinations of clinical and biochemical parameters could be used to predict mortality in COVID-19 patients. In a work by Halasz et al., a machine learning approach that used 6 clinical and biochemical features resulted in mortality prediction with an AUROC of 0.78 [0.74-0.84] (37). Zhao et al. presented a model using 7 clinical and biochemical variables which resulted in mortality prediction with AUROC of 0.83 [0.73-0.92] consistent with our results (38). Using machine learning algorithms with an input of 21 clinical or biochemical variables, Banoei et al. presented an in-hospital mortality prediction with AUROC of 0.91-0.95 (39). Thus, we acknowledge that our transcriptomic-only based approach yields predictive metrics that are in the same ranges of other published approaches. However, we propose the use of a tool that captures the immune profile of a patient directly through processing blood samples without added laborious hands-on time and resource. In regard, none of the elegant transcriptomic-based machine learning models which were previously described to predict mortality in COVID-19 (21, 23) can be easily implemented at the patient´s bedside due to constraints with RNAs processing and standardization of the measures. Similarly, while demographic parameters such as age or clinical parameters such as the SOFA score can readily be obtained at patient admission, some other variables used to build models in the literature are not so easily accessed. Going further, with regards to risk factors largely described in COVID-19 and observed in our study, adding age in the model improved mortality prediction with an AUROC of 0.84. Implementation of the IPP prototype device, that could be considered for use in clinical routine, may help in rapidly identifying patients at higher risk of death in order to provide early aggressive intensive care.

Although patients were enrolled in 5 different ICU in university hospitals (multi-center study), all ICUs are located within the same city (Lyon, France). This constitutes the main limitation of this study. Results need thus to be confirmed in cohorts from other cities/countries.

In conclusion, we showed that the multiplex transcriptomic panel prototype termed Immune Profiling Panel (IPP) could capture the dysregulation of immune responses of ICU COVID-19 patients at admission. Nine transcripts were associated with mortality in univariate analysis and this 9-mRNA signature remained significantly associated with mortality in a multivariate analysis including usual clinical confounders. Upon clinical/analytical validation and clearance by regulatory agencies, such fully automated and standardized immune monitoring tool could be used in clinical routine settings to quickly identify patients with higher risk of death requiring thus early aggressive intensive care.

## Data availability statement

The original contributions presented in the study are included in the article/Supplementary Material. Further inquiries can be directed to the corresponding author.

## Ethics statement

The studies involving human participants were reviewed and approved by Comité de Protection des Personnes Ile de France 1 – N°IRB/IORG #: IORG0009918. The patients/participants provided their written informed consent to participate in this study.

## Author contributions

GM, KB-P, and FV conceived the study, supervised the work and the analysis of all data. CT performed the majority of experiments and analyzed the data. VC, EP, MBu, and EC performed part of the IPP experiments. KI, MBo, and SB assisted with statistics and machine leaning analyses. A-CL, MBu, HY, MC, LA, M-CD, FW, FD, and CM helped for samples and data collection. A-CL, LK, and J-FL provided clinical inputs. FC and MG assisted with investigation. CT wrote the manuscript with inputs from GM. All authors discussed the results and commented on the manuscript. All authors contributed to the article and approved the submitted version.

## Funding

This work was supported by funds from the Hospices Civils de Lyon, Fondation HCL and Claude Bernard Lyon 1 University/Région Auvergne Rhône-Alpes and by bioMérieux. The funder was not involved in the study design, collection, analysis, interpretation of data, the writing of this article or the decision to submit it for publication.

## Conflict of interest

CT, VC, EC, KI, KB-P, EP, MBo, LK, SB, and J-FL are bioMérieux’s employees. EP, GM, and FV are co-inventors in patent applications covering the following markers: CX3CR1, CD127, IL10 and S100A9. bioFire – a bioMérieux company - holds patents on the technology. This does not alter the authors’ adherence to all the policies on sharing data and materials.

The remaining authors declare that the research was conducted in the absence of any commercial or financial relationships that could be construed as a potential conflict of interest.

## Publisher’s note

All claims expressed in this article are solely those of the authors and do not necessarily represent those of their affiliated organizations, or those of the publisher, the editors and the reviewers. Any product that may be evaluated in this article, or claim that may be made by its manufacturer, is not guaranteed or endorsed by the publisher.
